# Untargeted serum metabolomic profiling for early detection of *Schistosoma mekongi* infection in mouse model

**DOI:** 10.3389/fcimb.2022.910177

**Published:** 2022-08-18

**Authors:** Peerut Chienwichai, Kathyleen Nogrado, Phornpimon Tipthara, Joel Tarning, Yanin Limpanont, Phiraphol Chusongsang, Yupa Chusongsang, Kanthi Tanasarnprasert, Poom Adisakwattana, Onrapak Reamtong

**Affiliations:** ^1^ Princess Srisavangavadhana College of Medicine, Chulabhorn Royal Academy, Bangkok, Thailand; ^2^ Department of Molecular Tropical Medicine and Genetics, Faculty of Tropical Medicine, Mahidol University, Bangkok, Thailand; ^3^ Mahidol Oxford Tropical Medicine Research Unit, Faculty of Tropical Medicine, Mahidol University, Bangkok, Thailand; ^4^ Centre for Tropical Medicine and Global Health, Nuffield Department of Clinical Medicine, University of Oxford, Oxford, United Kingdom; ^5^ Department of Social and Environmental Medicine, Faculty of Tropical Medicine, Mahidol University, Bangkok, Thailand; ^6^ Department of Helminthology, Faculty of Tropical Medicine, Mahidol University, Bangkok, Thailand

**Keywords:** schistosomiasis, metabolomics, heptadecanoyl ethanolamide, picrotin, theophylline

## Abstract

Mekong schistosomiasis is a parasitic disease caused by blood flukes in the Lao People’s Democratic Republic and in Cambodia. The standard method for diagnosis of schistosomiasis is detection of parasite eggs from patient samples. However, this method is not sufficient to detect asymptomatic patients, low egg numbers, or early infection. Therefore, diagnostic methods with higher sensitivity at the early stage of the disease are needed to fill this gap. The aim of this study was to identify potential biomarkers of early schistosomiasis using an untargeted metabolomics approach. Serum of uninfected and *S. mekongi*-infected mice was collected at 2, 4, and 8 weeks post-infection. Samples were extracted for metabolites and analyzed with a liquid chromatography-tandem mass spectrometer. Metabolites were annotated with the MS-DIAL platform and analyzed with Metaboanalyst bioinformatic tools. Multivariate analysis distinguished between metabolites from the different experimental conditions. Biomarker screening was performed using three methods: correlation coefficient analysis; feature important detection with a random forest algorithm; and receiver operating characteristic (ROC) curve analysis. Three compounds were identified as potential biomarkers at the early stage of the disease: heptadecanoyl ethanolamide; picrotin; and theophylline. The levels of these three compounds changed significantly during early-stage infection, and therefore these molecules may be promising schistosomiasis markers. These findings may help to improve early diagnosis of schistosomiasis, thus reducing the burden on patients and limiting spread of the disease in endemic areas.

## Introduction

Schistosomiasis is one of the most common neglected tropical diseases harming global populations, especially underserved residents of African and South American countries (McManus et al., 2018). One report has estimated that approximately 800 million people are at risk of developing the disease, leading to at least 250 million infections and 280,000 deaths every year (Anisuzzaman and Tsuji, 2020). Schistosomiasis has two forms, intestinal and urogenital schistosomiasis, both caused by six species of blood fluke in the genus *Schistosoma*. Intestinal schistosomiasis is caused by *Schistosoma mansoni, S. japonicum, S. intercalatum, S. guineensis*, and *S. mekongi*. Patients with intestinal schistosomiasis may develop abdominal pain in the right upper quadrant, diarrhea, fatigue, and malaise. Advanced disease leads to splenomegaly, portal hypertension, and esophageal varices (McManus et al., 2018; Wilson, 2020),. In contrast, *S. haematobium* is the only species that causes urogenital schistosomiasis. Patients with urogenital schistosomiasis may show signs of pelvic pain, dysuria, and hematuria. In some chronic cases, urinary bladder cancer may occur secondary to chronic inflammation and tissue damage (McManus et al., 2018; Santos et al., 2021).

Currently, the standard method for diagnosing schistosomiasis is detection of eggs in feces or urine. However, damage may have already occurred to the patient’s body by the time eggs are detected. In addition, the sensitivity of this method is less than 50% (McManus et al., 2018). To overcome these limitations, many laboratory techniques have been employed to try to discover more sensitive diagnostic methods. For example, detection of circulating anodic and cathodic antigens (CAA and CCA) of *Schistosoma* spp. in patient samples is an interesting approach that has been tested in clinical settings. However, this test can show false positive results with certain patient conditions and the price is still too high to be economically viable (van Dam et al., 2013; Ogongo et al., 2018; McManus et al., 2018). Other diagnostic methods, such as detection of parasite DNA or serological testing, are available but these methods have limitations, particularly with their timeliness (McManus et al., 2018; Ogongo et al., 2018). Therefore, discovery of diagnostic markers with high sensitivity that can detect early-stage infection would help reduce the burden of this disease.

Mekong schistosomiasis is caused by *S. mekongi*. This parasite is common near the Mekong River, particularly in Laos and Cambodia (Muth et al., 2010). Despite the fact that *S. mekongi* is not found worldwide, it affects residents of endemic areas, immigrants, and visitors. Patients with Mekong schistosomiasis can be asymptomatic for years after infection during which time the parasite may be very active (Clerinx and van Gompel, 2011). Similar to other forms of schistosomiasis, diagnostic tests for Mekong schistosomiasis still have low sensitivity. The Kato-Katz technique is the most extensively used approach for the diagnosis of schistosomiasis. However, it has low sensitivity in low- and medium-intensity infections (Bergquist et al., 2009). Many approaches have been used in attempts to discover biomarkers of early *Schistosoma* infection.

Metabolomics is one fascinating approach for identifying potential biomarkers. Metabolomics is the qualitative and quantitative study of small molecules in biological samples (Fernández-García et al., 2018). It is widely used to study various aspects of schistosomiasis, for example, to elucidate the parasite’s response to drug treatments (Guidi et al., 2020), or to identify new drug targets (Chienwichai et al., 2021). Urine is an easily available and valuable body fluid for biomarker detection. For biomarker discovery, García-Pérez et al. (2008); Garcia-Perez et al. (2010a); Garcia-Perez et al. (2010b) performed a series of metabolomic studies using urine from *S. mansoni*-infected mice to identify the metabolomic fingerprint of the parasite. They found that the metabolites of infected mice were significantly different from those of control mice after 30 days of infection. Additionally, a number of small molecules, including phenyl acetyl glycine and glycolic acid, were identified as potential diagnostic markers (García-Pérez et al., 2008; Garcia-Perez et al., 2010a; Garcia-Perez et al., 2010b).

Similarly, Balog et al. (2011) analyzed urine samples from 447 individuals who lived in Uganda, an endemic area of *S. mansoni*. Their research found that infected people had been altered metabolites in gut microflora, energy metabolism, and liver function. In addition, Adebayo et al. (2018) studied the metabolomic fingerprint in serum and urine samples from *S. haematobium*-infected patients in Nigeria. The researchers found decreased levels of metabolites involved in chorismate production and increased levels of metabolites involved in choline and sphingolipid metabolism in infected patients. The study results also proposed catechol estrogens as a marker for advanced *S. haematobium* infection. Hu et al. (2020) combined metagenomic sequencing of gut microbiota with metabolomic data to explore the relationship between the intestinal microbiome and *S. japonicum* infection, as well as to identify novel markers of schistosomiasis. They found significant changes in the gut microbiome and in many metabolites with diagnostic potential, such as phosphatidylcholine, colfosceril palmitate, and naphthalenesulfonic acid.

Unfortunately, none of the aforementioned studies were able to identify markers of early infection with *Schistosoma* spp. Li et al. (2011) attempted to discover biomarkers of early *S. mansoni* infection in plasma, urine, and fecal samples from mice. The researchers collected biological samples from day 1 prior to infection to day 72 post-infection. However, the earliest metabolic changes that they found were on day 41 post-infection. Their data identified phenyl acetyl glycine in urine and 5-aminovalerate in feces as potential markers of infection. Currently, the study by Huang et al. (2020) is the only published report to detect *S. japonicum* infection as early as 1 week post-infection, which is 3 weeks faster than standard methods. This study found 17 serum metabolites from mice that correlated with progression of schistosomiasis, including diphenol glucuronide and glycerol tribenzoate. In our study, we employed a different strategy to screen for biomarkers of early *S. mekongi* infection in a mouse model. We collected serum metabolites at 2, 4, and 8 weeks post-infection and analyzed the metabolites with untargeted metabolomics and bioinformatic tools to screen for compounds with diagnostic potential. The aim of this study was to identify potential serum markers for early *S. mekongi* infection in mice using metabolomics. We hope that the findings of this study will be an initial step in the development of future point-of-care diagnostics for people with schistosomiasis.

## Materials and methods

### Ethics statement

Animal experiments were conducted in accordance with the guidelines for the use of animals at the National Research Council of Thailand (NRCT). All procedures were approved by the Faculty of Tropical Medicine Animal Care and Use Committee (FTM-ACUC), Mahidol University (Approval number: FTM-ACUC 015/2021).

### Animal maintenance, *S. mekongi* infection, and sample collection

Mice (*Mus musculus*) and freshwater snails (*Neotricula aperta*) were used to maintain *S. mekongi* in the laboratory as previously described (Phuphisut et al., 2018). Snails were collected from their natural habitat, the Mekong River, and cultured at the Applied Malacology Laboratory, Department of Social and Environmental Medicine, Faculty of Tropical Medicine, Mahidol University, Bangkok, Thailand. Ten 8-week-old female ICR mice were purchased from the National Laboratory Animal Center, Mahidol University and housed in controlled conditions at the Animal Care Unit, Faculty of Tropical Medicine, Mahidol University. Each mouse was infected with 25–30 *S. mekongi* cercariae. Blood was collected from the submandibular vein before infection and 2, 4, and 8 weeks after infection. Approximately 200 µL of blood was allowed to clot and then serum was collected after centrifugation at 2,000 g for 10 min at 4°C. Serum samples were stored at −80°C until further use. In addition, feces were collected from every mouse at each time-point for parasitological examination of eggs. At the end of the study, mice were euthanized using carbon dioxide gas.

### Quantification of *S. mekongi* eggs

Mouse stool samples were collected before infection and 2, 4, and 8 weeks after infection and processed for egg quantification using the modified Kato-Katz method (Katz et al., 1972).

### Metabolite extraction

Extraction of metabolites from serum was performed following a previously described protocol (Lu et al., 2014). In brief, 20 μL serum was mixed with 80 μL cold methanol and vortexed for 1 minute. This mixture was then incubated at 4°C for 20 minutes and centrifuged at 12,000 rpm for 10 minutes. Next, the supernatant was collected and dried with a speed vacuum (Tomy Digital Biology, Tokyo, Japan). Samples were stored at −80°C until further analysis.

### Metabolite identification by mass spectrometry

Ultra-high performance liquid chromatography (UHPLC; Agilent 1260 Quaternary pump, Agilent 1260 High Performance Autosampler and Agilent 1290 Thermostatted Column Compartment SL, Agilent Technologies, CA, USA) coupled to a quadrupole time-of-flight mass spectrometer (Q-TOF-MS) (TripleTOF 5600+, SCIEX, US) with electrospray ionization (ESI) using a DuoSpray ion source was used for metabolomic identification. For UHPLC separation, 0.1% formic acid in water was used as mobile phase A and 0.1% formic acid in acetonitrile was used as mobile phase B. A mixture of mobile phase A and B at a ratio of 50:50 (vol/vol) was used to resuspend samples and this mixture was transferred to a liquid chromatography (LC) vial for injection. LC vials were kept in the auto-sampler at 6°C during the analysis. Five microliters of sample was injected into the UHPLC with C18 reversed phase column (ACQUITY UPLC BEH, 2.1 × 100 mm, 1.7 µM, Waters). The flow rate for UHPLC was set to 0.3 mL/min at 40°C. The UHPLC-Q-TOF-MS system, mass ion chromatogram, and mass spectra were acquired by Analyst Software version 1.7 (SCIEX). The Q-TOF-MS was operated in positive (+ESI) and negative (-ESI) electrospray ionization modes. Data acquisition was performed with an information-dependent acquisition mode composed of a TOF-MS scan and 10 dependent product ion scans were used in the high sensitivity mode with dynamic background subtraction. The mass range of the TOF-MS scan was m/z 100–1,000 and the product ion scan was set to m/z 50−1,000. Equal aliquots of each metabolite sample were pooled to form the quality control (QC) samples. The QC samples were injected before, during, and after sample analysis to assess the system performance.

### Metabolite annotation with MS-DIAL

Metabolomic data from LC-MS/MS (.wiff and.wiff.scan files) were converted into a suitable file format (.abf files) using Reifycs Abf (Analysis Base File) Converter (https://www.reifycs.com/AbfConverter/). Metabolite annotation was performed with the MS-DIAL platform, which was developed by Tsugawa et al. (2015). MS-DIAL version 4.70 (http://prime.psc.riken.jp/compms/msdial/main.html) was used to annotate identified features by setting the parameters for soft ionization with chromatography separation type. The method used for MS was data-dependent metabolomics with profiling for both MS1 and MS/MS. Mass accuracies were 0.01 Da and 0.025 Da for MS1 and MS2, respectively. Peaks with a height of more than 1,000 amplitude and a mass slice width more than 0.1 Da were detected. The sigma window value for deconvolution was set to 0.5 and an MS/MS abundance cut off was not set. Metabolomic data was searched against all public MS/MS metabolite databases in positive mode (all public MS/MS: 290,915 records) and negative mode (all public MS/MS: 36,848 records). Retention time tolerance was set to 100 minutes and accurate mass tolerance was set at 0.01 and 0.05 for MS1 and MS2, respectively. The cut off for identification score was 80%. All adduct ions were included in the study. Retention tolerance and MS1 tolerance for the alignment parameters setting were 0.05 minutes and 0.015 Da, respectively.

### Pathway analysis

Pathways containing metabolites that changed significantly after infection were identified using the STITCH database version 5.0 [http://stitch.embl.de/: (Szklarczyk et al., 2016)]. A list of altered metabolites from each time-point were uploaded and Mus musculus was selected as the organism of origin. The minimum required interaction score was set to medium confidence and no more than 10 interactions were shown for each node.

### Potential biomarker enrichment

The method for identifying potential biomarkers used two modules of the Metaboanalyst online platform version 5.0 [https://www.metaboanalyst.ca/: (Pang et al., 2021)], “Statistical analysis” and “Biomarker analysis”. For both modules, metabolite names along with their concentrations were explored in a time-series analysis with one factor. Metabolomic data were filtered by interquartile range. Moreover, data were normalized with quantile normalization, which is suitable for datasets with more than 1,000 features. Data were transformed with cube root transformation and scaled with range scaling. Visualization was performed using correlation coefficients with Pearson’s analysis, feature importance detection with random forest, and receiver operating characteristic (ROC) curve analysis.

### Statistical analysis

Statistical analysis was performed using built-in feature of MS-DIAL and SPSS Statistics for Windows version 15.0 (SPSS Inc., Chicago, IL, USA). Metabolites altered more than 1.5-fold with a p-value less than 0.01 were considered significant. ANOVA was used to compare metabolite levels in each sample.

## Results

### Distribution of metabolomic data

**Figure 1:**
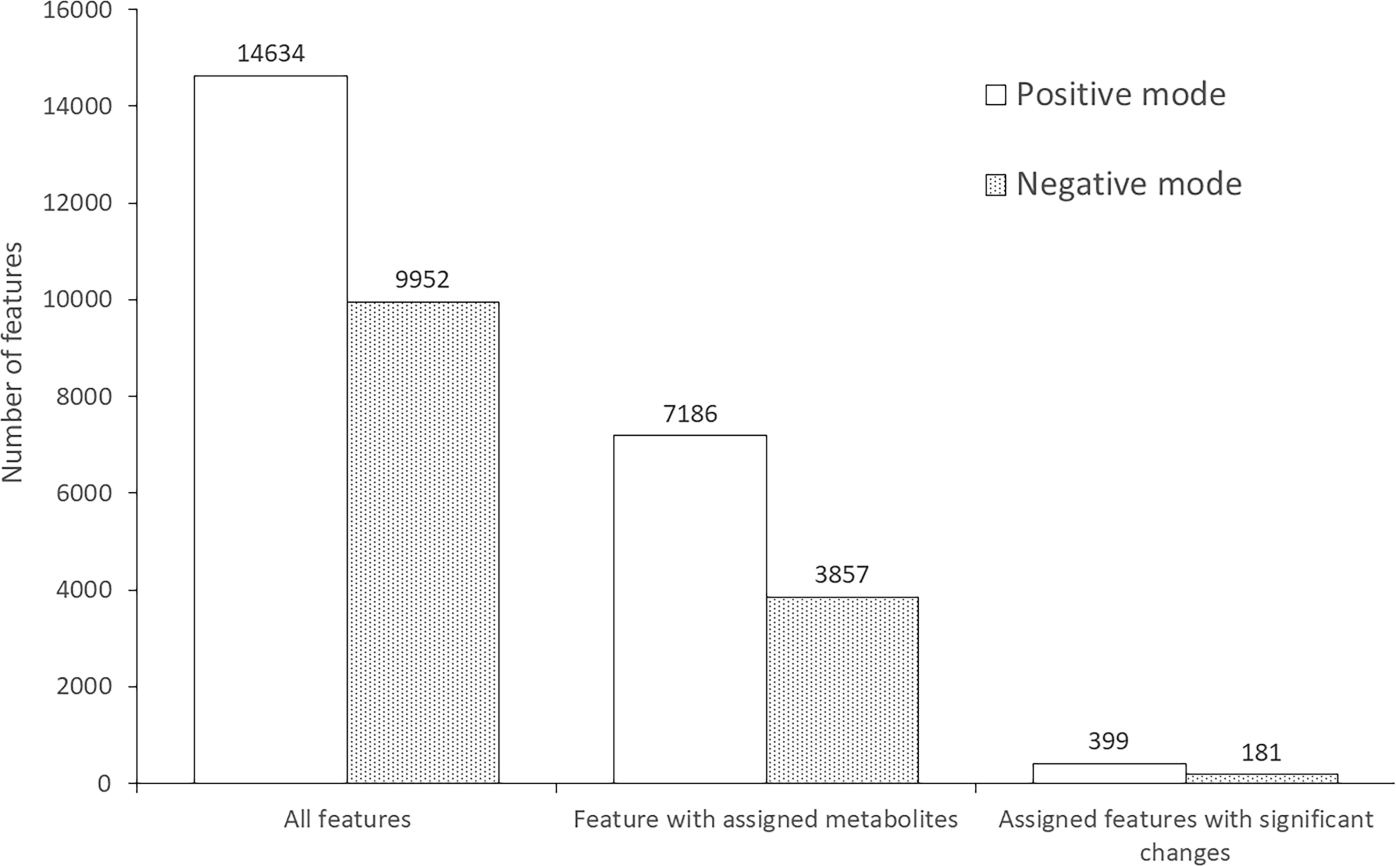
Number of features identified from metabolomic analysis of mouse serum after *S. mekongi* infection.

From all conditions, metabolomic analysis identified a total of 24,586 features, including 14,634 features in positive mode (**Supplementary Table S1**) and 9,952 features in negative mode (**Supplementary Table S2**). Approximately half of the features were annotated with metabolites in the database: 7,186 and 3,857 features for positive and negative mode, respectively. Interestingly, the level of 399 positive-mode features and 181 negative-mode features were significantly different after *S. mekongi* infection, as shown in **Figure 1**.

**Figure 2:**
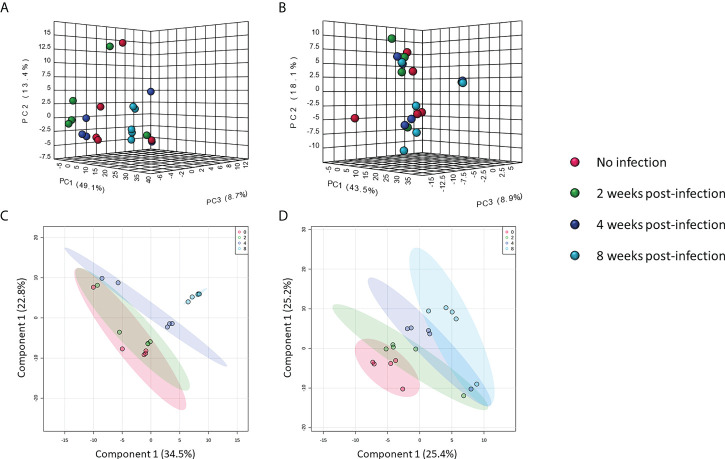
Multivariate analysis of metabolomic data. Interactive 3D-PCA of features in positive mode **(A)** and negative mode **(B)**. PLS-DA of features in positive mode **(C)** and negative mode **(D)**.

Metabolomic data were further analyzed with multivariate statistics using unsupervised interactive three-dimensional principal component analysis (3D-PCA) and supervised partial least squares discriminant analysis (PLS-DA). Interactive 3D-PCA analysis of features in the positive mode showed differences among pre-infection samples and samples from other time-points (**Figure 2A**). Differences were not observed in features in the negative mode (**Figure 2B**). Notably, PLS-DA showed clear clustering of samples from features in both positive and negative mode. Each cluster was separated from no infection over time (**Figures 2C, D**), indicating effects of infection on compound moieties in mouse serum.

### Serum metabolites changed prior to parasitological detection of parasite eggs from feces

No *S. mekongi* eggs were observed in mouse feces 2 and 4 weeks after infection. It was not until 8 weeks after infection that 575 eggs/g feces were identified. Therefore, diagnosis by fecal analysis for parasite eggs was only possible 8 weeks after infection. In contrast, using metabolomic analysis of mouse sera, significant differences in serum metabolites were detected as early as 2 weeks after *S. mekongi* infection. There were 76 metabolites with significantly altered levels at 2 weeks post-infection, whereas no eggs were detected in the mouse feces at this time-point (**Table 1**; **Supplementary Table S3**). N-arachinoyl-5-hydroxytryptamide, lapachol, and (1S)-1,5-anhydro-1-[4,5,10-trihydroxy-2-(hydroxymethyl)-9-anthryl]-D-glucitol were the most differentially expressed metabolites with 153.77-, 153.00- and 141.75-fold change, respectively. At 4 weeks post-infection, there were 91 significantly altered metabolites; however, no eggs were detected in the mouse feces at this time-point (**Table 1**; **Supplementary Table S4**). Lapachol, gibberellin A9, and 5-hydroxy-2,2-dimethyl-10-(2-methylbut-3-en-2-yl)pyrano[3,2-g]chromen-8-one were the metabolites with the most significant changes (191.41-, 41.83-, and 34.05-fold change, respectively). Parasite eggs were first detected in feces at 8 weeks post-infection. At this time-point, 359 metabolites were significantly differentially expressed (**Table 1**; **Supplementary Table S5**). At 8 weeks post-infection, 1-Phenanthrenecarboxylic acid, 1,2,3,4,4a,9,10,10a-octahydro-1,4a-dimethyl-7-(1-methylethyl)-9-oxo-, 8-{(1S,5R)-4-Oxo-5-[(2Z)-2-penten-1-yl]-2-cyclopenten-1-yl}octanoic acid, and kahweol were the most affected metabolites with 39.77-, 31.88-, and 31.42-fold change, respectively.

**Table 1:** Top 10 metabolites with the highest fold change at 2, 4, and, 8 weeks after *S. mekongi* infection.

No.	Metabolite name	Adducted form	Mode	Retention time	m/z	Fold -change	*P*-value
Top-10 metabolites with highest fold change from 2 weeks after infection							
1	N-arachinoyl-5-hydroxytryptamide	[M+H]^+^	Positive	11.71657	471.396	153.77	0.0003
2	Lapachol	[M-H]^-^	Negative	8.346058	241.0877	153.00	0.009
3	(1S)-1,5-anhydro-1-[4,5,10-trihydroxy-2-(hydroxymethyl)-9-anthryl]-D-glucitol	[M+3H]^+^	Positive	13.6515	441.1258	141.75	0.003
4	Glabrol	[M+H]^+^	Positive	13.93778	415.1875	133.63	0.002
5	13-dodecan-2-yl-6-(1-hydroxyethyl)-3-(hydroxymethyl)-12-methyl-9-propan-2-yl-1-oxa-4,7,10-triazacyclotridecane-2,5,8,11-tetrone	[M+H]^+^	Positive	15.37448	559.4083	116.04	2.61E^-07^
6	Onopordopicrin	[M+2ACN+H]^+^	Positive	14.36326	366.1922	114.70	0.0009
7	5-[5-hydroxy-3-(hydroxymethyl)pentyl]-8a-(hydroxymethyl)-5,6-dimethyl-3,4,4a,6,7,8-hexahydronaphthalene-1-carboxylic acid	[M+H-2H2O]^+^	Positive	13.09474	377.2274	100.77	0.004
8	3,5,7,8-tetramethoxy-2-(3,4,5-trimethoxyphenyl)chromen-4-one	[M+K]^+^	Positive	14.59533	471.1048	98.22	0.0001
9	3,5,7,8-tetramethoxy-2-(3,4,5-trimethoxyphenyl)chromen-4-one	[M+2Na-H]^+^	Positive	14.66728	471.1013	93.95	0.004
10	3-Buten-2-one, 4-[4-(beta-D-glucopyranosyloxy)-2-hydroxy-2,6,6-trimethylcyclohexylidene]-	[M+H]^+^	Positive	14.59533	409.1857	86.94	8.54E^-05^
Top-10 metabolites with highest fold change from 4 weeks after infection							
1	Lapachol	[M-H]^-^	Negative	8.346058	241.0877	191.41	0.009
2	Gibberellin A9	[M-H]^-^	Negative	7.290267	315.1576	41.83	0.008
3	5-hydroxy-2,2-dimethyl-10-(2-methylbut-3-en-2-yl)pyrano[3,2-g]chromen-8-one	[M-H]^-^	Negative	7.796092	311.1892	34.05	0.005
4	Picrotin	[M+FA-H]^-^	Negative	1.181697	309.0999	30.00	9.57E^-12^
5	Picrotin	[M+CH_3_ COONa-H]^-^	Negative	6.826533	309.1021	22.35	1.44E^-06^
6	1-Phenanthrenecarboxylic acid, 1,2,3,4,4a,9,10,10a-octahydro-9-hydroxy-1,4a-dimethyl-7-(1-methylethyl)-, (1S,9R)-	[M+Li]^+^	Positive	8.561492	299.201	22.13	0.005
7	Phosphatidylserine	[M-H]^-^	Negative	8.783392	808.5076	14.02	0.009
8	Fragilin	[M+Br]^-^	Negative	1.185329	317.014	14.02	4.23E^-11^
9	2,3,4’-Trihydroxy-4-Methoxybenzophenone	[M+Na-2H]^-^	Negative	1.181697	259.0627	12.12	3.01E^-08^
10	4-[(2S)-2-hydroxy-3-methyl-3-[(2S,3R,4S,5S,6R)-3,4,5-trihydroxy-6-(hydroxymethyl)oxan-2-yl]oxybutoxy]furo[3,2-g]chromen-7-one	[M-H]^-^	Negative	10.49793	447.1328	10.87	0.009
Top-10 metabolites with highest fold change from 8 weeks after infection							
1	1-Phenanthrenecarboxylic acid, 1,2,3,4,4a,9,10,10a-octahydro-1,4a-dimethyl-7-(1-methylethyl)-9-oxo-	[M+H]^+^	Positive	8.729832	315.197	39.77	4.13E^-11^
2	8-{(1S,5R)-4-Oxo-5-[(2Z)-2-penten-1-yl]-2-cyclopenten-1-yl}octanoic acid	[M+H]^+^	Positive	8.8087	315.1931	31.88	0.008
3	Kahweol	[M+Na]^+^	Positive	8.7304	337.1788	31.42	2.64E^-08^
4	GR 113808	[M+H]^+^	Positive	13.60072	394.1867	30.34	1.79E^-08^
5	[(E)-3-acetyloxy-7-hydroxy-6-methoxy-7-(6-oxo-2,3-dihydropyran-2-yl)hept-4-en-2-yl] acetate	[M+H]^+^	Positive	8.14756	395.0924	30.12	1.27E^-11^
6	Progesterone	[M-H]^-^	Negative	10.83639	313.2149	24.88	0.0099
7	Epiandrosterone	[M+2ACN+H]^+^	Positive	10.4684	255.2101	22.24	5.37E^-09^
8	Acetamide, N-[(Z)-2-(acetylamino)-1-[(7-methoxy-1,3-benzodioxol-5-yl)methyl]ethenyl]-N-(2-oxo-3-phenylpropyl)-	[M+2K-H]^+^	Positive	10.44938	439.1872	21.34	1.82E^-06^
9	(2S,3S,4S,5R,6R)-6-(3-benzoyloxy-2-hydroxypropoxy)-3,4,5-trihydroxyoxane-2-carboxylic acid	[M+H]^+^	Positive	8.258067	395.0909	17.12	0.002
10	Norethindrone	[M-C_6_H_10_O_4_+H]^+^	Positive	8.572973	299.2005	16.39	7.46E^-13^

"+" positively charged ion, "−" negatively charged ion.

**Figure 3:**
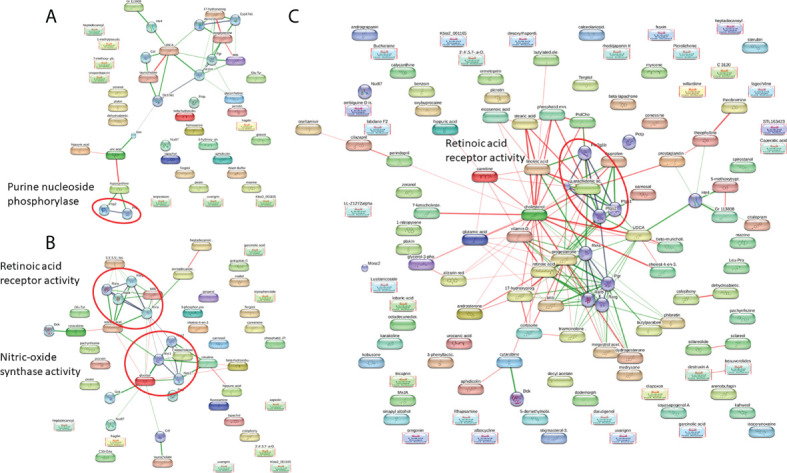
Pathway analysis of significantly altered metabolites at 2 weeks **(A)**, 4 weeks **(B)**, and 8 weeks post-infection **(C)**.

To gain more understanding into the metabolomic changes that occur after *S. mekongi* infection, we performed pathway analysis of the altered metabolites to identify potential metabolic impacts (**Figure 3**). At the 2-week time-point after infection, the purine nucleoside phosphorylase pathway was enriched with a false discovery rate of 0.00587 (**Figure 3A**). Interestingly, the retinoic acid receptor activity pathway was highlighted in both the 4- and 8-week post-infection data set with false discovery rates of 2.72e^-06^ and 0.048, respectively (**Figures 3B, C**).

### Heptadecanoyl ethanolamide, picrotin, and theophylline are potential biomarkers for early infection with *S. mekongi*

**Figure 4:**
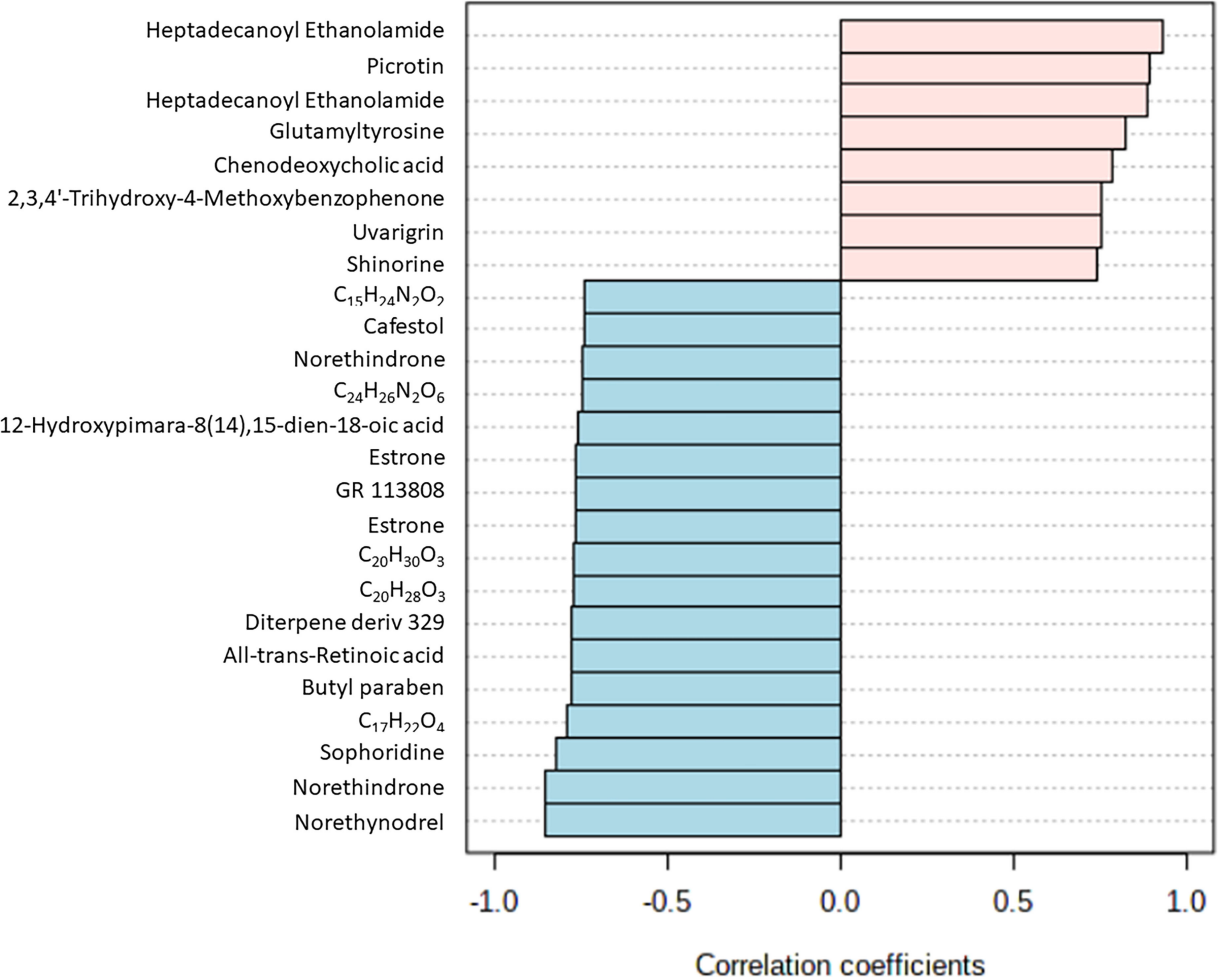
Top 25 metabolites with highest/lowest correlation coefficient from Pearson’s correlation test.

**Figure 5:**
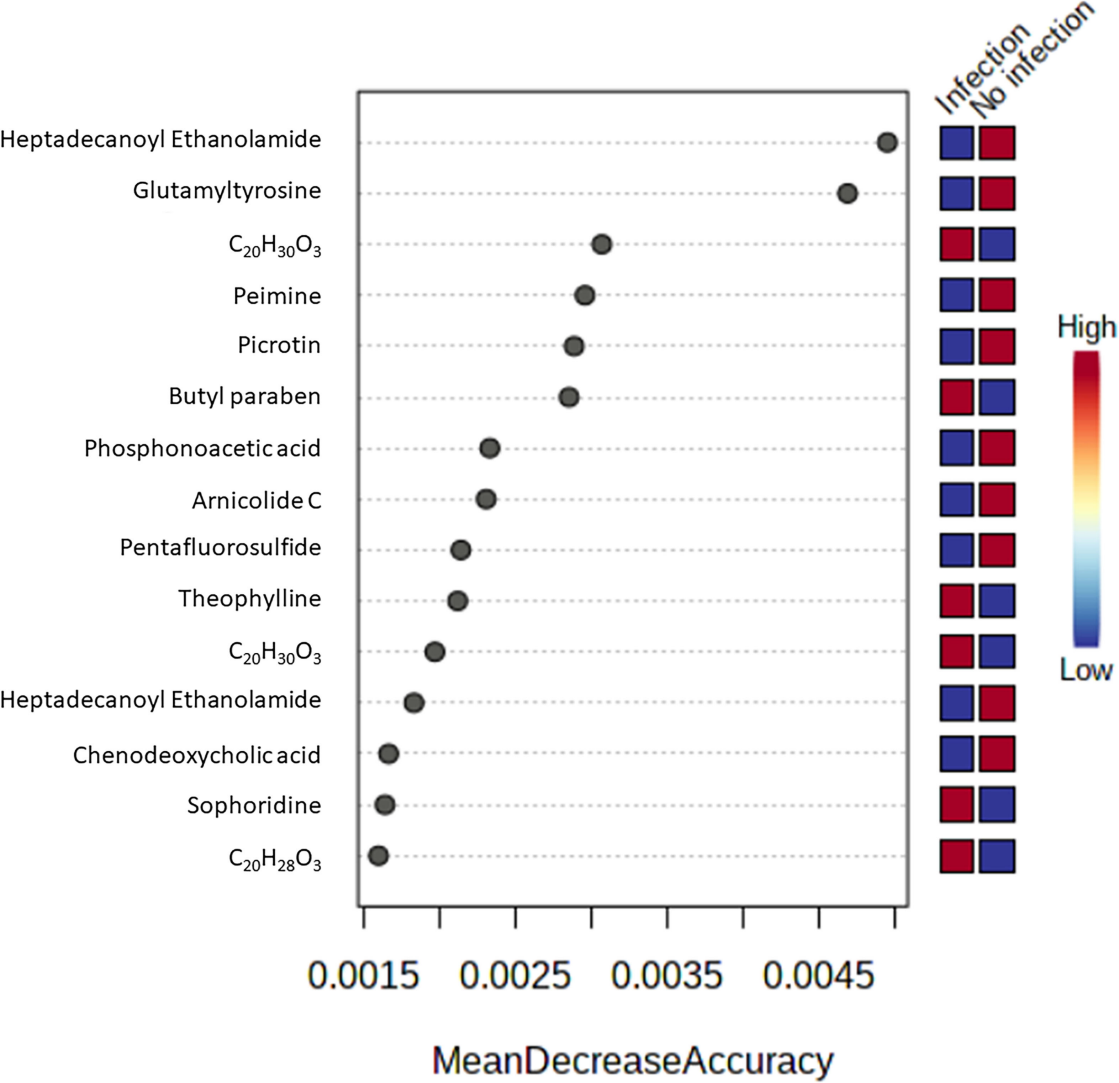
Top 15 metabolites with highest mean decrease accuracy from feature important detection with random forest algorithm.

Biomarker screening of metabolomic data was performed using three approaches: correlation coefficient analysis, feature important detection, and receiver operating characteristic (ROC) curve analysis. Correlation coefficient analysis with Pearson’s test showed 116 metabolites that had significant positive or negative correlation with infection duration and a *p-*value less than 0.01 (**Figure 4**; **Supplementary Table S6**). Norethynodrel, norethindrone, and sophoridine were the most significantly up-regulated metabolites. Heptadecanoyl ethanolamide ([M+H]^+^), picrotin, and heptadecanoyl ethanolamide ([M+2H+Na]^+^) were the most significantly down-regulated metabolites. In addition, feature important detection with random forest algorithm was performed to select important features with biomarker potential. The mean decrease accuracy was calculated and 167 metabolites with significantly altered expression were identified (**Figure 5**; **Supplementary Table S7**). C_20_H_30_O_3_, butyl paraben, and theophylline were the most significantly up-regulated metabolites, whereas hepadecanoyl ethanolamide, glutamyltyrosine, and peimine were the most significantly down-regulated metabolites. ROC curve analysis was also used to screen the diagnostic ability of the identified metabolites. A total of 117 metabolites showed significance at one or more time-points after *S. mekongi* infection (**Supplementary Table S8**). Theophylline, all-trans-retinoic acid, and GR 113808 were the most up-regulated metabolites. In contrast, picrotin, heptadecanoyl ethanolamide ([M+H]^+^), and heptadecanoyl ethanolamide ([M+2H+Na]^+^) were the most significantly down-regulated metabolites. After integrating the three biomarker screening approaches, we identified four metabolites that were significantly differentially expressed at 2, 4, and 8 weeks after *S. mekongi* infection (**Table 2**).

**Table 2:** Metabolites identified as significant in ROC curve analysis at all infection time-points.

No.	Metabolite name	2-weeks post-infection			4-weeks post-infection			8-weeks post-infection		
		AUC	t-test	Log_2_ Fold change	AUC	t-test	Log_2_ Fold change	AUC	t-test	Log_2_ Fold change
1	Picrotin	1	0.0009	2.24	1	2.4141E^-05^	4.91	1	6.1898E^-06^	4.27
2	Heptadecanoyl Ethanolamide ([M+H]^+^)	1	0.0004	1.82	1	0.0002	2.48	1	1.1391E^-05^	2.65
3	Heptadecanoyl Ethanolamide ([M+2H+Na]^+^)	1	0.0004	1.75	1	0.0004	2.54	1	0.0002	2.45
4	Theophylline	0.92	0.006	-0.73	0.92	0.009	-0.94	1	0.0005	-0.89

**Figure 6:**
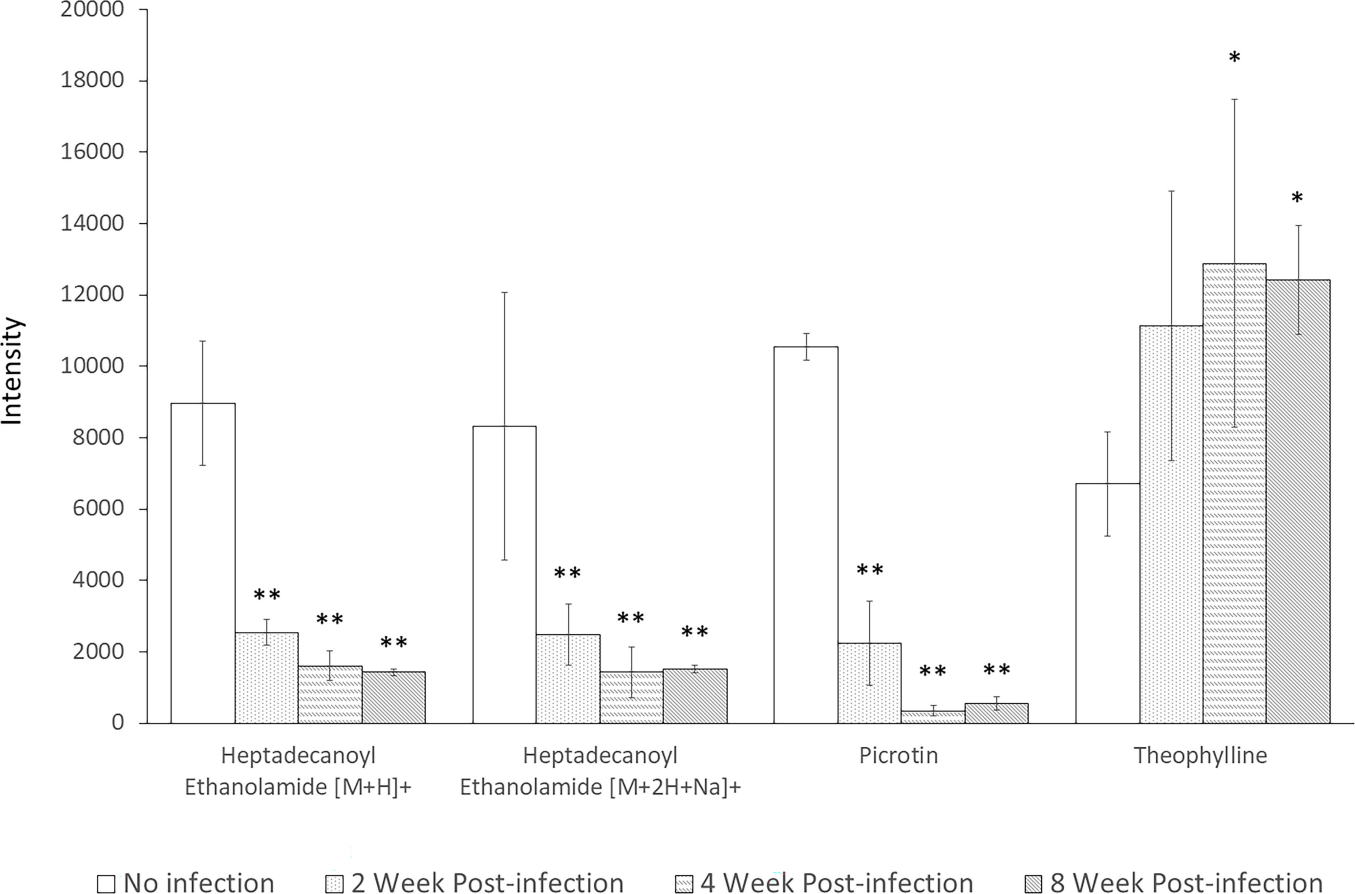
Levels of Heptadecanoyl Ethanolamide with [M+H]+ adduct, Heptadecanoyl Ethanolamide with [M+2H+Na]+ adduct, Picrotin, and Theophylline at all time-points. * indicates statistical significance with *p*-value < 0.05. ** indicates statistical significance with *p*-value < 0.01.

Interestingly, these four metabolites were identified as significantly altered in all screening approaches. These metabolites were heptadecanoyl ethanolamide with [M+H]^+^ adduct, heptadecanoyl ethanolamide with [M+2H+Na]^+^ adduct, picrotin, and theophylline. The levels of both adducted forms of heptadecanoyl ethanolamide and of picrotin were significantly decreased after infection at all time-points with *p-*values less than 0.01. In contrast, the levels of theophylline were significantly increased at 4 and 8 weeks post-infection (**Figure 6**). Therefore, these results suggest that heptadecanoyl ethanolamide, picrotin, and theophylline could be potential biomarkers for early-stage schistosomiasis.

## Discussion

Schistosomiasis is a major parasitic disease that affects many global populations. Early detection can reduce disease progression and complications. Untargeted metabolomics is a high-throughput approach that systematically evaluates the levels of large numbers of compounds in biological samples. Metabolomics has been used to discover new biomarkers for many diseases. In this study, we used metabolomics to screen for biomarkers of early *S. mekongi* infection. Serum from infected mice was collected at 2, 4, and 8 weeks post-infection and was analyzed with mass spectrometry. The resulting metabolomic data were then compared bioinformatically to data from pre-infection samples.

Biomarkers discovery in early schistosomiasis is the crucial step for reducing clinical illness of patients as well as preventing disease transmission. Pathogenesis of *Schistosoma* infection occurs from immunologic reaction of host body to parasite’s eggs, which detection of the disease prior to egg production apparently protect patients from the disease (McManus et al., 2018). In the past few decades, many types of biomolecules have been identified as potential markers, for example, DNA (Aula et al., 2021), proteins (Zhou et al., 2010; Grenfell et al., 2013), and metabolites (Li et al., 2011; Huang et al., 2020). To date, several attempts have been made to identify metabolite biomarkers at the early stage of schistosomiasis. Li et al. (2011) tried to detect significantly altered metabolites in the serum, urine, and feces of *S. mansoni*-infected mice. They found altered metabolites at day 41 after infection. Recent findings from Huang et al. (2020) identified significantly altered metabolites from 1 week post-infection with *S. japonicum*. Huang et al. (2020) found 73 potential biomarkers of schistosomiasis and 55 metabolites with altered expression during the 1–2 weeks following infection. In our study, we identified 76 metabolites with altered levels after 2 weeks of *S. mekongi* infection. One limitation of our study was that the first time-point was 2 weeks, not 1 week, after infection. However, our study and the Huang et al. study identified similar metabolites, such as hypoxanthine, inosine and 1-methylinosine, arachidonic acid and N-arachinoyl-5-hydroxytryptamide.

Hypoxanthine is a molecule involved in purine metabolism and is essential for nucleic acid synthesis. Increased levels of hypoxanthine can be found in many infectious diseases, including onchocerciasis (Bennuru et al., 2017), malaria (Chen et al., 2019), and tuberculosis (Conde et al., 2021). Notably, altered hypoxanthine levels were also detected in the serum of *S. haematobium*-infected children (Osakunor et al., 2020). Therefore, changes in hypoxanthine levels may reflect pathogenic infections, including with *Schistosoma* spp.

Our study identified a 114.70-fold change in onopordopicrin at 2 weeks post-infection with *S. mekongi*. This metabolite was previously reported to have anti-inflammatory activities (de Almeida et al., 2013). Therefore, it may be involved in the immune-escape mechanism used by *Schistosoma* parasites.

Our study also found a 14.02-fold change in phosphatidylserine (PS) at 4 weeks post-infection. PS can trigger its receptors and cause phagocytes to produce anti-inflammatory and immunosuppressive responses. Parasite-derived phosphatidylserine is a key molecule for evading host defense mechanisms (Wanderley et al., 2020). Therefore, *S. mekongi* might produce PS for host immune evasion purposes.

Progesterone was another differentially expressed metabolite that we identified at 8 weeks post-infection. Progesterone induces evagination and growth of *Taenia solium* cysticerci in humans (Escobedo et al., 2010). In addition, the frequency of *T. solium* cysticercosis in pigs is higher during pregnancy, which is characterized by a large increase in progesterone levels (Bazer et al., 2008). Therefore, the increased progesterone in *S. mekongi*-infected mouse sera might support growth of the parasite.

Pathway analysis was performed to better understand the metabolic changes after *S. mekongi* infection. The differentially expressed metabolites at each time-point showed significant pathway alterations. At 2 weeks after infection, metabolites associated with the purine nucleoside phosphorylase pathway were increased (**Figure 3A**). The purine nucleoside phosphorylase pathway is a part of purine metabolism and converts inosine into hypoxanthine or guanosine into guanine and ribose-1-phosphate (D'Muniz Pereira et al., 2011). *Schistosoma* worms lack a *de novo* purine synthesis pathway; therefore, acquiring these molecules from the host is essential for parasite survival (Angelucci et al., 2011; D'Muniz Pereira et al., 2011). Interestingly, alteration of purine-related metabolism was also observed in a metabolomic study on serum from preschool-aged children infected with *S. haematobium* (Osakunor et al., 2020). At 4 and 8 weeks after infection, retinoic acid receptor activity was highlighted as a major affected pathway (**Figures 3B, C**). The retinoic acid receptor activity pathway is part of the retinoic acid signaling pathway that controls development and plays fundamental roles in the physiology of chordate animals. Retinoic acid binds to its nuclear receptor and initiates many downstream genetic events (Das et al., 2014; Ghyselinck and Duester, 2019). The effects of blood fluke infection on the host retinoic acid signaling pathway are still unclear. However, retinoic acid serum levels were significantly higher in *S. mansoni*-infected multiple sclerosis patients than in uninfected patients or healthy controls. In addition, genes involved in the biosynthesis and metabolism of retinoic acid were induced in dendritic cells. These findings indicate that *Schistosoma* infection might interfere with host retinoic acid production (Correale and Farez, 2013).

Our study integrated three approaches to identify potential serum biomarkers of early-stage schistosomiasis: (i) correlation coefficient analysis; (ii) feature important detection; and (iii) ROC curve analysis. First, correlation coefficient analysis can identify compounds with altered levels at different time-points (Xia et al., 2012) and is commonly used to identify potential biomarkers in metabolomic data (Tian et al., 2016; Tang et al., 2021). The second analytic method, feature important detection with random forest algorithm, is a decision tree-based statistical machine learning method that can be used to identify potential biomarkers from omics data (Acharjee et al., 2020). This algorithm has been previously used to screen for serum markers in *Onchocerca volvulus* infection (Denery et al., 2010). The third method, ROC curve analysis, is an approach designed for selection of potential biomarkers that considers the trade-off between the sensitivity and specificity of features of interest (Chong et al., 2019). Several studies have used ROC curve analysis to identify markers of parasitic infections, including *Toxocara canis* and *Toxoplasma gondii* infection (Zhou et al., 2016; Zheng et al., 2019). From our data set containing 11,043 annotated features, data processing with correlation coefficient analysis, feature important detection, and ROC curve analysis identified 116, 167, and 117 potential serum markers, respectively (**Supplementary Tables S3**-**S5**). By comparing results from the three different approaches, we found four overlapping features, which were two adducted forms of heptadecanoyl ethanolamide, picrotin, and theophylline. These three compounds were differentially expressed at 2, 4, and 8 weeks post-infection and therefore, they may be potential serum biomarkers for early-stage Mekong schistosomiasis. However, only limited information is available on the role of these three metabolites in parasitic diseases. More studies are needed to explore their roles in the host and parasite.

Heptadecanoyl ethanolamide is a saturated fatty acid with 17 carbon atoms conjugated with ethanolamide (McGurk et al., 2021). This molecule is a member of the N-acyl ethanolamide class of bioactive lipid species. In biological systems, N-acyl ethanolamide is derived from cell membranes and further converted into fatty acids and ethanolamide. Lipids in the N-acyl ethanolamide class are involved in inflammation and immunity (McGurk et al., 2021). Heptadecanoyl ethanolamide can be found in various tissues and biological samples, including liver, skin, serum, and feces (Kendall et al., 2019; Jackson et al., 2020; Chen et al., 2021). Moreover, this metabolite is involved in skin inflammation after exposure to ultraviolet radiation (Kendall et al., 2019). A derivative of heptadecanoyl ethanolamide, 1-heptadecanoyl-sn-glycero-3-phosphocholine, has also been shown to be down-regulated 1 week after *Toxoplasma gondii* infection (Zhou et al., 2016). In our study, a reduction in heptadecanoyl ethanolamide levels was found after *S. mekongi* infection. Because parasites can manipulate the host immune system (Cooke, 2012) and heptadecanoyl ethanomide is associated with skin inflammation, *S. mekongi* may mediate the reduction of this inflammation-related molecule to protect itself from host immunity.

Picrotin is another potential *S. mekongi* infection biomarker identified in this study. Picrotin acts as a Gamma–aminobutyric acid (GABA) receptor stimulant and convulsant that has been extensively used to induce seizures in neurological studies (Li and Slaughter, 2012; Fan et al., 2019; Melo-Carrillo et al., 2020). Some anthelmintic medicines act rapidly and selectively on nematode neuromuscular transmission (Mendonça-Silva et al., 2004; Callau-Vázquez et al., 2018). Levamisole, pyrantel, and morantel are nicotinic acetylcholine receptor agonists that cause spastic paralysis in nematode muscle. Organophosphorus cholinesterase antagonists include dichlorvos and haloxon. Piperazine is a GABA agonist that causes flaccid paralysis in worm muscles (Martin, 1997). Therefore, *Schistosoma* parasites may reduce picrotin in mouse sera to prevent over-stimulation of GABA receptors, which could lead to muscle paralysis.

Theophylline levels also increased after *S. mekongi* infection. This molecule is part of the caffeine metabolism pathway and is known for its bronchodilator effect (Ohmichi et al., 2018). Changes in serum theophylline levels have been found in several conditions, such as exhaustive exercise, systemic lupus erythematosus-related nephritis, and Parkinson’s disease (Li et al., 2017; Ohmichi et al., 2018; Shi et al., 2020). Regarding metabolism of theophylline in schistosomiasis patients, some patients have shown reduction of theophylline elimination from their bodies (Cukier et al., 1992). This finding may relate to the increased theophylline levels that we found in mouse sera after *S. mekongi* infection.

Several markers are high potential to diagnose pan-schistosome infection. The CCA and CAA are good examples of conserved antigen among *Schistosoma* species. The CCA/CAA-based diagnostic tests can detect all species of human schistosomiasis (Corstjens et al., 2020; Homsana et al., 2020). Due to their close phylogeny, many vital genes of the parasites in this genus are conserved, for example, Acetylcholinesterase (Bentley et al., 2003), G protein-coupled receptors (Campos et al., 2014). According to this similarity, there is possibility that the highlighted metabolites from *S. mekongi* infection might extrapolate other *Schistosoma* infection. However, further studies need to be explored to compare candidate metabolite markers of *S. mekongi* infection with other parasitic infections and other illnesses.

In summary, we performed untargeted metabolomics on serum from *S. mekongi*-infected mice at three time-points—2, 4, and 8 weeks post-infection—and compared infected serum with serum from uninfected mice. Three different approaches were used to select potential markers of *S. mekongi* infection. Heptadecanoyl ethanolamide, picrotin, and theophylline were highlighted as potential biomarkers of early-stage schistosomiasis and remained altered throughout the course of the study. These findings may help facilitate early diagnosis of Mekong schistosomiasis. However, the specificity of these biomarkers to *S. mekongi* infection needs to be further studied. Moreover, the differential expression of heptadecanoyl ethanolamide, picrotin, and theophylline in other infectious diseases should be further investigated for evaluating the specificity to Mekong schistosomiasis.

## Data availability statement

The original contributions presented in the study are included in the article/**Supplementary Material**. Further inquiries can be directed to the corresponding author.

## Ethics statement

Animal experiments were conducted in accordance with the guidelines for the use of animals at the National Research Council of Thailand (NRCT). All procedures were approved by the Faculty of Tropical Medicine Animal Care and Use Committee (FTM-ACUC), Mahidol University (Approval number: FTM-ACUC 015/2021).

## Author contributions

All authors participated in the design and interpretation of the study; PCi and KN analyzed the results; PT, JT, and OR conducted the metabolomics experiments; YL, PCg, YC, KT, and PA prepared parasites; PCi and OR performed bioinformatics analysis; All authors wrote, revised, and approved the final manuscript.

## Funding

This work was supported by an Innovation Project grant (69864), a New Discovery and Frontier Research Grant FY2022 and a ICTM grant from Mahihol university awarded to OR. This research project was also supported by Chulabhorn Royal Academy to PCi and international postdoctoral fellowships awarded by Mahidol University to KN and OR.

## Conflict of interest

The authors declare that the research was conducted in the absence of any commercial or financial relationships that could be construed as a potential conflict of interest.

## Publisher’s note

All claims expressed in this article are solely those of the authors and do not necessarily represent those of their affiliated organizations, or those of the publisher, the editors and the reviewers. Any product that may be evaluated in this article, or claim that may be made by its manufacturer, is not guaranteed or endorsed by the publisher.

## Supplementary material

Supplementary Table S1 Metabolites identified in positive mode from the serum of uninfected and infected mice at different time-points.

Supplementary Table S2 Metabolites identified in negative mode from the serum of uninfected and infected mice at different time-points.

Supplementary Table S3 Metabolites with the highest fold change at 2 weeks after *S. mekongi* infection.

Supplementary Table S4 Metabolites with the highest fold change at 4 weeks after *S. mekongi* infection.

Supplementary Table S5 Metabolites with the highest fold change at 8 weeks after *S. mekongi* infection.

Supplementary Table S6 Potential biomarkers of schistosomiasis identified by correlation coefficient analysis.

Supplementary Table S7 Potential biomarkers of schistosomiasis identified by feature important detection with random forest algorithm.

Supplementary Table S8 Potential biomarkers of schistosomiasis identified by ROC curve analysis.
